# Solitary primary intraosseous xanthoma of the mandible in a 15-year-old boy: a case report

**DOI:** 10.1186/s13256-024-04534-y

**Published:** 2024-05-07

**Authors:** A. Georgiev, S. Genova, P. Uchikov, Krasimir Kraev, M. Kraeva, D. Chakarov, A. Uchikov

**Affiliations:** 1 Department of Maxillo Facial Surgery, Multiprofile Hospital for Active Medical Treatment “Sv. Panteleimon” Plovdiv, Plovdiv, Bulgaria; 2 Department of General and Clinical Pathology, Medical Faculty, Medical University of Plovdiv, Plovdiv, Bulgaria; 3 Department of Special Surgery, Medical Faculty, Medical University of Plovdiv, Plovdiv, Bulgaria; 4 Department of Propedeutics of Internal Diseases, Medical Faculty, Medical University of Plovdiv, 15A Vasil Aprilov Boulevard, Plovdiv, 4000 Bulgaria; 5 Department of Otorhinolaryngology, Medical Faculty, Medical University of Plovdiv, Plovdiv, Bulgaria; 6 Department of Propaedeutics of Surgical Diseases, Section of General Surgery, Faculty of Medicine, Medical University of Plovdiv, 4002 Plovdiv, Bulgaria

**Keywords:** Xanthoma, Mandible, 15-years-old boy, Case report

## Abstract

**Background:**

A xanthoma is a rare bone condition consisting of a predominant collection of lipid-rich, foamy histiocytes. The central xanthoma of the jaws is a unique benign tumor.

**Case report:**

A 15-year-old Caucasian male has been presented to our department. He had radiological changes in the area of the left mandibular angle, with an area of diffuse osteolysis of 3.0 cm by 2.0 cm. Computed tomography reveals an area of diffuse osteolysis that starts from the distal root of the lower second molar and reaches the ascending process. A bone biopsy was performed, which revealed a benign proliferative process composed of histiocytic cells involving and infiltrating trabecular bone in a background of loose fibrous connective tissue devoid of any other significant inflammatory infiltrate. The size of the formation was 2.9 cm by 2.0 cm. Immunohistochemical staining for CD68 was strongly positive and negative for S-100 and CD1a. From routine blood tests, cholesterol, triglycerides, and blood sugar are within normal values, which excludes systemic metabolic disease. Subsequent to the surgical intervention, the patient underwent postoperative assessments at intervals of 14, 30, 60 days, and a year later, revealing the absence of any discernible complications during the aforementioned observation periods.

**Conclusion:**

The diagnosis of primary xanthoma of the mandible is rare and can often be confused with other histiocytic lesions. A differential diagnosis should be made with nonossifying fibroma and Langerhans cell histiocytosis, as in our case. In these cases, immunohistochemistry with CD 68, S-100, and CD1a, as well as blood parameters, are crucial for the diagnosis.

## Background

A xanthoma is a rare soft tissue and bone condition consisting of a predominant collection of lipid-rich, foamy histiocytes. Lesions have been described in the axial and appendicular bones in patients with and without hyperlipidemia [1].

Heretofore, a xanthomatous predilection for the jaws has been associated with Hand–Schüller–Christian disease, or eosinophilic granuloma. However, xanthogranuloma represents a different clinical entity and is not related to either of those two conditions. This benign solitary bone lesion occurs independently of any other systemic sites of xanthomatous involvement [2].

The primary xanthoma of bone is extremely rare and, when present, is often secondary to hyperlipidemia type II or III or diabetes mellitus [2, 3]. When systemic metabolic disease and lipid disease are ruled out, the bony lesion is termed a primary xanthoma of bone [4].

Xanthomas, indicative of disrupted lipid metabolism, manifest as yellowish papules, plaques, or nodules characterized by lipid-laden macrophages (foam cells). Notably, xanthomas often serve as early signs of familial hypercholesterolemia, associating with an augmented risk of premature coronary artery disease and heart failure. The discussed case underscores the contemporary imperative of swift clinical assessments, particularly in identifying compound heterozygotes who may exhibit xanthomas early in life [5]. Emphasizing risk stratification and genetic counseling, current management strategies involve lifestyle adjustments and pharmacotherapies such as statins, bile acid sequestrants, fibrates, ezetimibe, or other lipid-modifying agents, with the potential for concurrent amelioration of xanthomas through addressing underlying dyslipidemia [6, 7].

## Case report

### Clinical data

A 15-year-old, Caucasian male went into a dental office for an ordinary clinical examination due to an upcoming orthodontic treatment. After radiography of the jaws, a lesion on the left side of the mandible angle was discovered. The following day, the boy was sent to the clinic of oral and maxillofacial surgery at “Sv. Panteleimon” hospital in Plovdiv for evaluation.

### Laboratory data

From routine blood tests, cholesterol, triglycerides, and blood sugar are within normal values. Total cholesterol was 4.28 mmol/l, low-density lipoprotein (LDL) cholesterol was 0.79 mmol/l, triglycerides were 1.06 mmol/l, and blood glucose was 5.27 mmol/l. The other indicators were without deviation from the normal range.

### X-ray

**Fig. 1:**
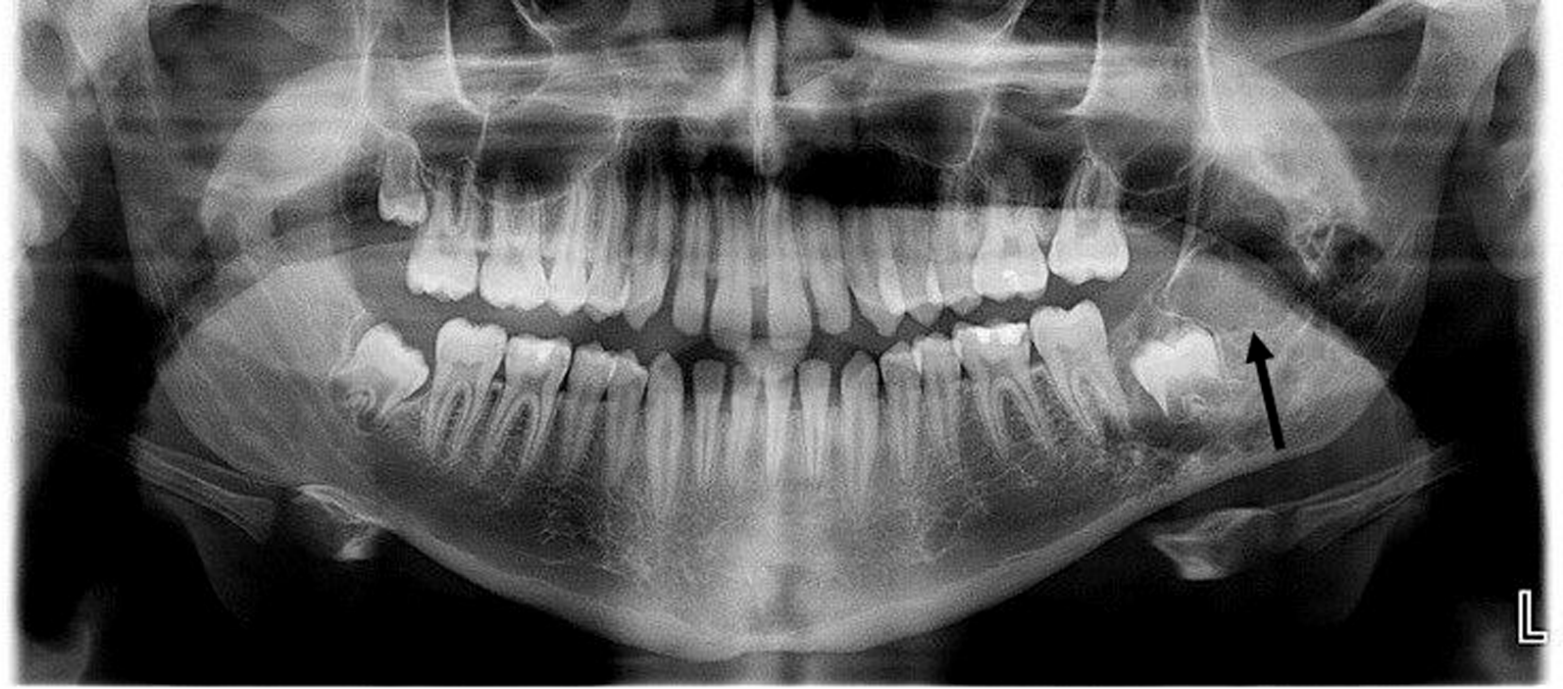
Panoramic radiograph on presentation. The area of diffuse lesion with uneven outlines and blurred boundaries of the surrounding normal bone (arrow)

An orthopontomography in the area of the left mandibular angle shows an area of diffuse lesion with uneven outlines and blurred boundaries of the surrounding normal bone (Fig. 1).

### Computed tomography (CT)

**Fig. 2:**
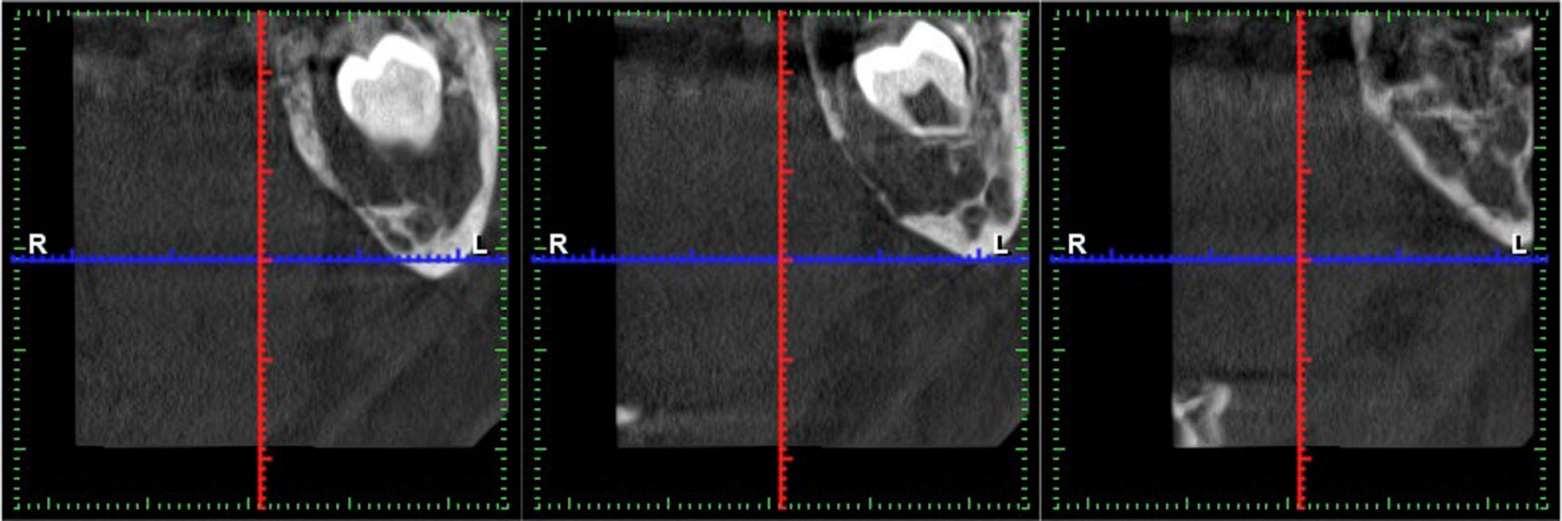
CT—the area of diffuse osteolysis in the left mandibular angle

**Fig. 3:**
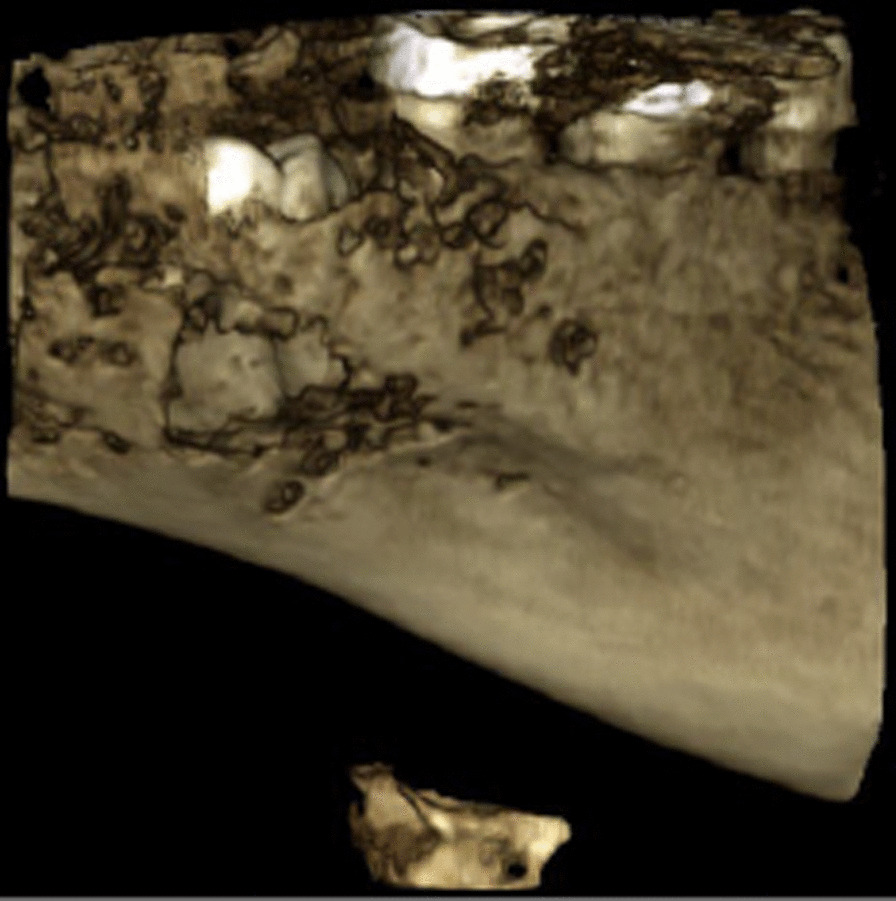
Loss of normal trabecular pattern in the medullary cavity

An area of diffuse osteolysis is observed on the axial computed tomography sections in the area of the left mandibular angle, which starts from the distal root of the lower second molar and reaches the ascending process. The mandibular canal is also involved in some places (Figs. 2, 3).

### First operation

**Fig. 4:**
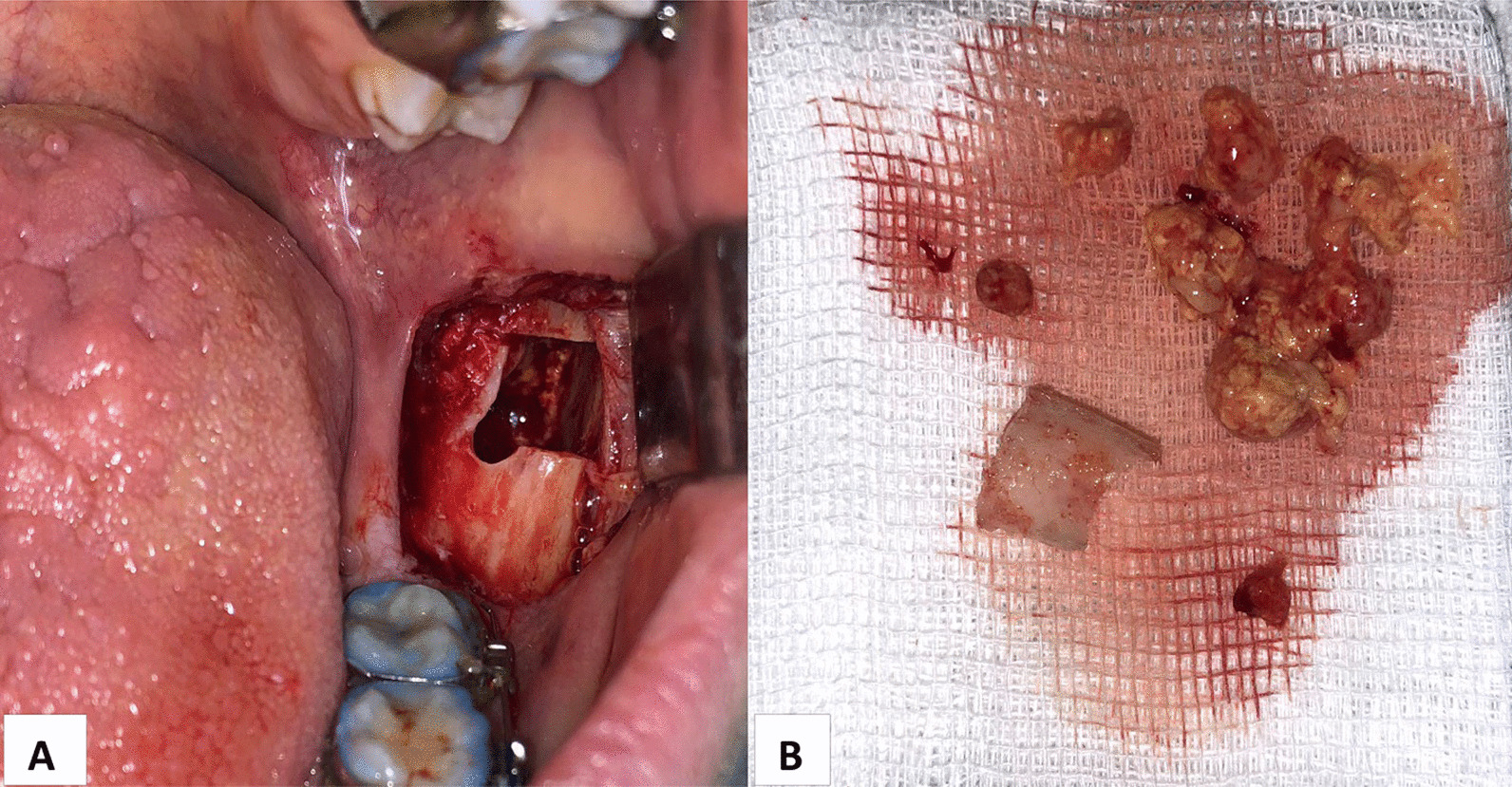
**A** Buccal cortical plate window. **B** Biopsy specimen—soft, yellow ‘‘granules’’

Three days after the initial diagnosis was established, a bone biopsy was performed via intraoral approach under local anesthesia. Access was made into the retromolar space by retracting a flap and removing a 3.0 cm by 2.0 cm buccal cortical plate window using a high-speed handpiece and a chisel. A tissue specimen was collected using a bone curette and a bone gauge. Yellow ‘‘granules’’ were clinically noted in the cancellous bone (Fig. 4).

### Histological and immunohistochemical examination

**Fig. 5:**
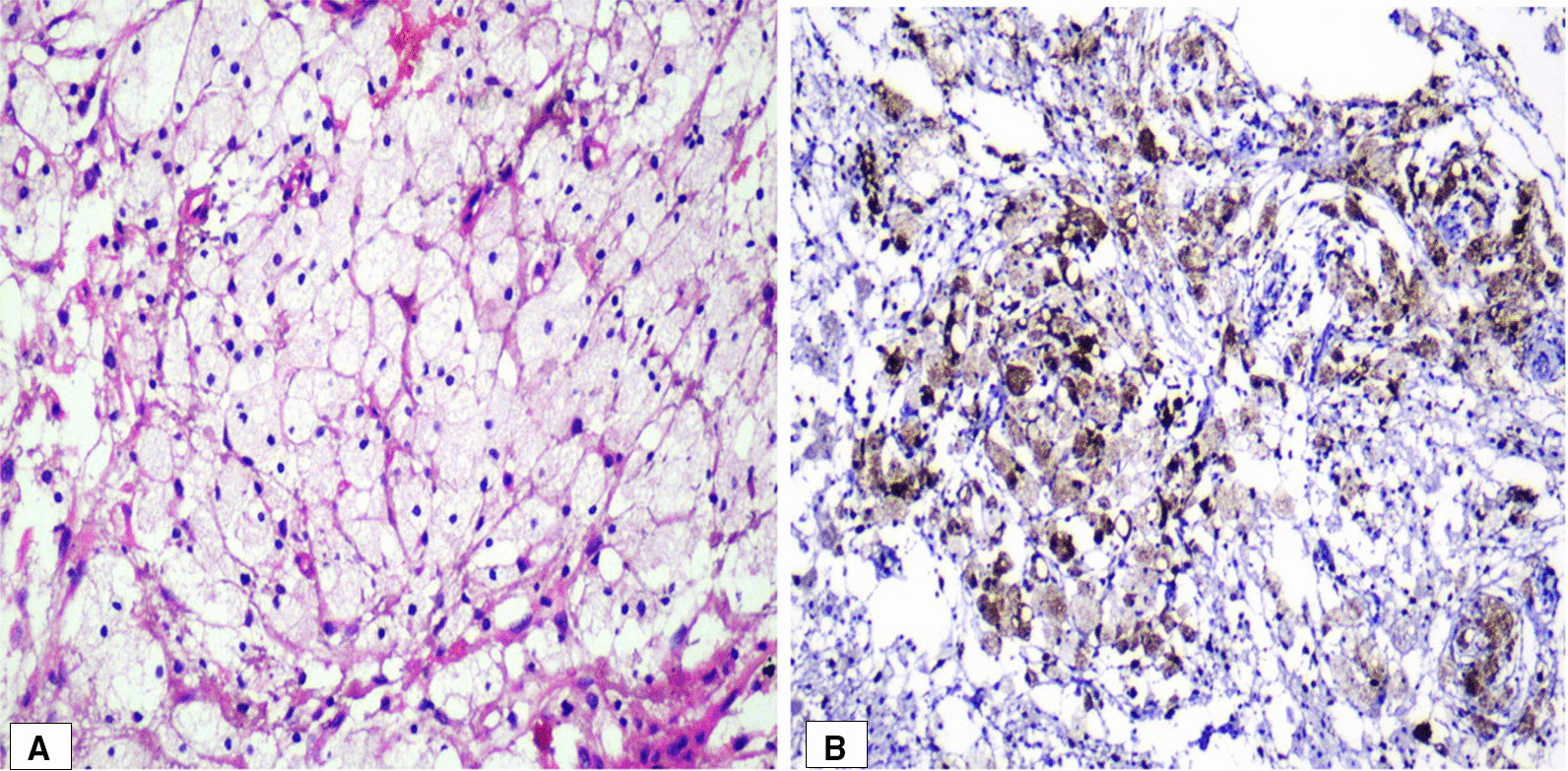
**A** Sheets of xanthoma cells with loose connective tissue stroma. Hematoxylin and eosisn (HE); original magnification, ×40. **B** Xanthoma cells, densely positive for CD68. Immunohistochemistry, ×20

The buccal cortical plate and specimen were submitted in 10% formalin for histologic examination, which showed a benign proliferative process composed of histiocytic cells involving and infiltrating trabecular bone in a background of loose fibrous connective tissue devoid of any other significant inflammatory infiltrate. High-power examination demonstrated an abundance of foamy cells (Fig. 5A).

Immunohistochemical staining was performed on formalin-fixed, paraffin-embedded 5-μm sections. This was achieved following antigen retrieval using citrate pH 6.0, blocking endogenous biotin and peroxidase. DAKO’s immunostainer was utilized for this procedure. Antibody binding to the cells in sections was detected using the horseradish peroxidase (HRP) reaction kits (DAKO, Carpinteria, CA, USA) according to the manufacturer’s protocol instructions. Images were visualized and captured with a digital camera mounted on a Nikon Eclipse 80i microscope using NIS-Elements Advanced Research Software version 4.13 (Nikon Instruments; Tokyo, Japan). Immunohistochemical staining for CD68 was strongly positive to confirm the histiocytic/macrophage lineage of the cells and negative for S-100 and CD1a, which excludes Langerhans cell-related histiocytic disease (Fig. 5B).

### Second operation

**Fig. 6:**
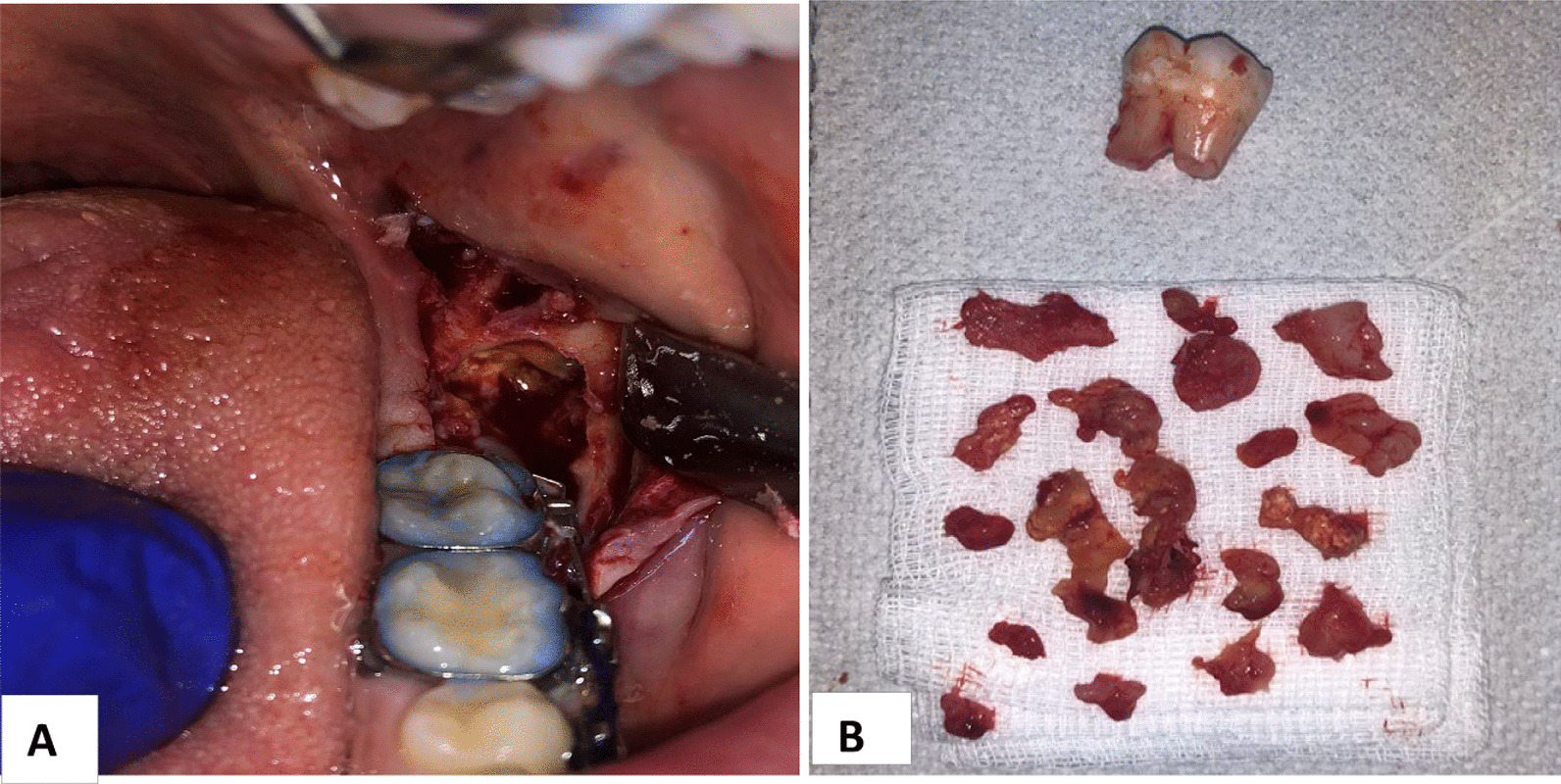
**A**, **B** Second operation—complete excochleation of the tumor

Under a combination of intravenous sedation and local anesthesia, a second operation was performed. Access was made by an initial horizontal incision in the retromolar space and a vertical incision in the left vestibule. After reflecting a triangle mucoperiostal flap, the bone was fenestrated in the retromolar area with burs. The third molar (wisdom tooth) was exposed and pulled out. The tumor tissue was removed with a curette. An osteoplasty of the cavity was performed with round burs. After replacing the flap it was sutured (Fig. 6A, B).

### Follow-up

After the surgical procedure, the patient underwent postoperative evaluations at 14-day, 30-day, and 60-day intervals, followed by an evaluation 1 year later. These evaluations indicated the absence of any noticeable complications throughout the specified observation periods.

## Discussion

Histiocytic diseases have been divided into Langerhans cell-related histiocytic disease (LCH) and a non-Langerhans histiocytic processes. The xanthoma is a non-Langerhans histiocytic process and is characterized microscopically by lipid-containing macrophages, or foamy cells [4, 8–10].

The primary xanthoma of bone is extremely rare and when systemic metabolic disease and lipid disease are excluded, the bony lesion is termed the primary xanthoma of bone [11–14].

Majority of cases are seen in the second and third decades of life. There is no gender predilection. Jaw lesions presented as solitary radiolucencies with a predilection for the posterior mandible. Unlike maxillary lesions, pain and expansion are inconsistent findings in mandibular lesions. Jaw lesions are not associated with extragnathic bone or soft tissue involvement or a hyperlipidemia. The central xanthoma of the jaws is a unique benign tumor. Histopathologically, many other jaw lesions contain variable numbers of foamy histiocytes. Therefore, a diagnosis of a central xanthoma of the jaws must be made after excluding all other histiocyte containing lesions [8, 15].

The diagnosis of primary xanthoma of the mandible is rare and can often be confused with other histiocytic lesions. The central xanthoma of the jaw bones is being recognized as an entity with features unique enough to warrant separation from conditions such as benign fibrous histiocytoma (BFH) and nonossifying fibroma (NOF), with which it is often confused [16, 17].

A total of 29 cases of primary/central xanthoma of the jaw bones have been described in the English literature. Jaw lesions were seen across a wide age range from 11 to 72 years, with a mean age of 33.95 years. However, 55% of cases (16 out of 29) were seen in the 2nd and 3rd decades of life. The xanthomas of the craniofacial bones average only a few centimeters in size. In the mandible, reported xanthomas range from 1 cm to 4 cm. The radiographic appearance may range from a small, well-demarcated radiolucent lesion with sclerotic margins to diffuse appearance with small ill-defined radiolucencies and areas of increased density [8, 11–16, 18–20].

Computed tomography shows loss of normal trabecular pattern in the medullary cavity, and the lesion may have a higher density than the normal bone marrow [15, 21].

Histopathologically, histiocytes and lipid-containing macrophages, or foamy cells, are present exhibiting a faintly granular cytoplasm, occasional multinucleated giant cells, and fibrous connective tissue. Cholesterol clefts can also be present. Immunohistochemical staining is used to differentiate the macrophage, or non-Langerhans histiocytic process, from the Langerhan’s histiocytosis. Xanthomas exhibit diffuse strong positive staining for CD 68 and are uniformly negative for S-100 and CD1a, in contrast to Langerhan’s cell histiocytosis, which is positive for S-100 and CD1a [4, 9, 10, 12]. Other disease processes that display xanthomatous presentations include Rosai–Dorfman disease, Erdheim–Chester disease, and Gaucher’s disease [8, 15].

Xanthomatous presentations should be considered on histological differential diagnosis, such as the nonossifying fibroma (NOF) and benign fibrous histiocytoma (BFH). Histologically, the two lesions are indifferentiable. The diagnosis is made according to clinical and radiological appearance [15, 22]. NOF usually affects the long bones [13, 17]. Histopathologically, the NOF and BFH shows spindle-shaped cells, foamy histiocytes, and multinucleated giant cells with a stromal tissue background in a storiform pattern, differentiating the two forms of the xanthoma [9, 15]. In our case, the fibrous lesions with focal ossification are mainly in the periphery, which makes it necessary to think in the direction of xanthofibroma, but the predominant picture is of xanthoma cells.

The central xanthoma of the jaws is characterized primarily by a proliferation of histiocytes. Based on the sampling of tissue, these findings are seen in a diverse list of conditions including periapical inflammatory lesions, benign fibrous histiocytoma, nonossifying fibroma of bone, and fibrous dysplasia [15].

Different theories regarding the pathogenesis of xanthoma exist. One theory suggests lipid leakage from vessels after local trauma or hemorrhage. Another theory suggests xanthomatous transformation of undifferentiated mesenchymal cells by lipotrophic factors in the blood in patients with autoimmune conditions [23]. After a histologic diagnosis of intrabony xanthoma is rendered, a lipid metabolic disorder work-up consisting of a full clinical and hematologic exam is necessary to exclude systemic endocrine or metabolic disease [8].

Primary intraosseous xanthoma of the mandible can be treated with curettage, and the prognosis is satisfactory even with only partial excision [11, 24]. When removed entirely by curettage, recurrence has not been reported. The long term prognosis is good, and malignant transformation has not been reported [8, 24]. In the mandible, spontaneous resolution has not been reported. Radical excision and chemotherapy are discouraged and radiation therapy may have limited therapeutic effect as the xanthoma may not be a true neoplastic disorder [24]. In our case 0.12 months after the operation, the patient is fully recovered and local recurrence after the operation is not observed.

In a broader context, comparing this case with existing literature reveals commonalities, such as the characteristic radiographic appearance and histopathological features of xanthomas [25–27]. However, the uniqueness of this case lies in its rare manifestation in the mandible, necessitating differentiation from other histiocytic lesions. Furthermore, the absence of systemic metabolic or lipid disorders underscores the rarity of primary xanthoma of bone. The case aligns with the prognosis of primary intraosseous xanthomas, emphasizing the efficacy of curettage as a treatment modality with a favorable long-term outcome. The discussion of pathogenesis theories and the importance of lipid metabolic disorder work-up post-diagnosis contributes to a broader understanding of xanthoma etiology and management. Overall, this case not only adds valuable clinical insights to the limited literature on primary xanthoma of the mandible but also highlights the success of a meticulous diagnostic and therapeutic approach in achieving a positive patient outcome.

## Conclusion

The diagnosis of primary xanthoma of the mandible is rare and can often be confused with other histiocytic lesions. A differential diagnosis should be made with NOF and Langerhan’s cell histiocytosis, as in our case. In these cases, immunohistochemistry with CD 68, S-100, and CD1a, as well as blood parameters are crucial for the diagnosis. Further investigations into the molecular basis of xanthomatous transformations and advancements in diagnostic tools are crucial for refining targeted therapeutic strategies. Addressing these aspects will contribute to overcoming current challenges and enhancing our understanding and treatment of xanthomas.

## Data Availability

Not applicable.
